# Therapeutical and Pharmaceutical Notes

**Published:** 1900-10

**Authors:** W. J. Martin

**Affiliations:** Kankakee, Ill.


### THERAPEUTICAL AND PHARMACEUTICAL NOTES.

UNDER THE DIRECTION OF W. J. MARTIN, M.D.C.,
KANKAKEE, ILL.

PARALDEHYDE AND CHLOROFORM FOR ANÆSTHETIC PURPOSES. The *New Orleans Medical and Surgical Journal* for March, 1900, contains an article on this topic by Noto. As a result of experiments on animals, he concludes that the association of paraldehyde with chloroform, owing to its effects, appears to be by far superior to all other methods of mixed anæsthesia proposed by experimenters up to this time. Administering paraldehyde before the inhalation of chloroform, first of all, we remove from the patients who are rebellious or fretful that consciousness of being forced to undergo the chloroform narcosis, and this in certain instances will prove a great practical advantage. The period of chloroform excitement, which often is much protracted and reaches great intensity, is completely suppressed. The profuse secretion of saliva, so disgusting to the patient and also to the operator, and which sometimes becomes dangerous, is done away with, and vomiting is not produced, at least judging from the observations he has been enabled to make thus far.

By previously administering paraldehyde the chloroform anæsthesia is obtained in a very short time, and with a very small dose of chloroform the sleep proceeds soundly, respiration continues calmly, the heart works on with perfect regularity, and the blood-pressure even in the deepest narcosis is strong enough.

Another fact not to be passed over is that with paraldehyde the sleep is much prolonged, even after the cessation of anæsthesia. The patient awakes some little time after the completion of the operation, already revived and without suffering the effects of trauma.

Before ending his memoir, Noto wishes to insist upon one point, with the hope that by having recourse to the association of paraldehyde with chloroform it should be made possible to practice anæsthesia without any danger even to those patients who are affected with heart-disease, and for whom the plain chloroform narcosis is absolutely contraindicated.

We know, indeed, that paraldehyde does not provoke trouble in such patients, and the quantity of chloroform necessary to produce anæsthesia in such cases becomes so small that all danger for the cardiovascular function is considered as avoided.—*Therapeutic Gazette.*

THE EFFECTS AND DANGERS OF CHLORETONE. Dr. R. D. Rudolf (*Canadian Practitioner*) summarizes the results of his investigations as follows: 1. Chloretone would seem, as has been found by Houghton and Aldrich, to be an ideal anæsthetic for physiological work. There might be some doubt about the recovery of the animals, however, and this would limit its use to cases in which recovery is not desired. The preliminary administration of chloroform or ether might be used here, but this increases the risk, of course. 2. It has little or no effect upon the pulse, respiration, and blood-pressure for hours, but eventually, if the dose be large enough, these become depressed and the animal dies, the heart stopping before respiration. 3. Chloretone has a most marked and depressing effect upon the body temperature, lowering this more than any other drug with which we are acquainted, with the possible exception of alcohol. This depressing effect is evident before the animal is even drowsy, and is in ratio to the dose given. It may be partially prevented by keeping the animal very warm. 4. Any drug which can exert such an effect on the total heat of the body is one which requires to be used with great caution in medical practice. This is doubly important, as the drug is very slowly gotten rid of, and we know of no antidote with the exception, perhaps, of external warmth.

HYPNOTICS ANTAGONISTIC TO COCAINE. Gioffredi has found (*Ber. d. Deut. Phar. Ges.*) that cocaine is completely antagonized by various hypnotics, including paraldehyde and the urethans, doses exceeding those otherwise fatal having been administered with impunity when followed by physiologic antidotes. Curious to relate, however, cocaine is not an antidote to the corresponding hypnotics.

LARGE DOSES OF CARBOLIC ACID IN EQUINE TETANUS. Place (*The Lancet*, February 24, 1900) adds an appendix to Atkinson's case of bubonic plague, published in the *Lancet* of December 9, 1899. He states that during the last two years he has been treating tetanus in horses by means of hypodermatic injections of carbolic acid. The mode of procedure is to inject in the neighborhood of the neck and shoulders one drachm of carbolic acid (*British Pharmacopœia*) every two hours for the first thirty-two hours of treatment, and less frequently as occasion may arise afterward. The result is that within an hour there is a large swelling at the seat of injection, which gradually subsides during convalescence, and there is no trace of it left after fourteen days in the case of the injections made during the virulence of the disease. Sometimes, however, in the case of the injections made during the subsidence of the disease, there is a loss of hair. This occasionally persists for weeks, but in no case has it been found to be permanent. The largest amount used in a single case has been thirty-six fluidrachms in eighty-four hours, the patient an Arab horse fourteen years old, which recovered and resumed work on the twenty-second day after the attack. Place has never administered less than sixteen drachms in a successful case, and states that, as in two cases he has seen horses die after the administration by the mouth of half an ounce of diluted carbolic acid given for pneumonia, he can only conclude that in tetanus there is a specific tolerance of the acid, and judging from Dr. Atkinson's case that the same seems to be the case in plague.—*Therapeutic Gazette.*

[We heartily indorse the hypodermatic use of carbolic acid in the treatment of equine tetanus, it having given us better results in combating this most fatal disease than any other single drug or combination of drugs which we have ever used. Our formulæ for administering carbolic acid hypodermatically is as follows: ℞.—Carbolic acid, 95 per cent., two ounces; glycerin puris, one ounce; distilled aqua, one ounce. Mix. Sig. Inject two drachms every two to four hours according to the severity of the case. These injections will cause blebs or puffy swellings of the skin, but these will subside gradually without loss of hair or other inconvenience to the animal. Of course, in the treatment of tetanus by carbolic-acid injections other proper therapeutic measures must be employed, such as the laying open and thorough curetting of the wound when this is practical, and afterward dressing antiseptically; the administering of enemas of warm water and glycerin to overcome constipation, a sloppy nutritious diet, and confining the animal to dark, quiet quarters.—W. J. M.]

In a monograph on the treatment of tetanus with carbolic acid, Dr. V. Ascoli, of Rome, Italy, states his conclusions as follows: "1. Statistics show better results from the carbolic method than from the use of serum. 2. The carbolic acid must be given hypodermatically and in large doses. 3. Under its influence the muscular contractions and spasms diminish to a marked degree. 4. The acid acts in tetanus particularly as an antitoxic and a moderator of reflex activity of the nerve centres. 5. The energetic local disinfection, combined with the support of the patient's strength, are the cardinal points in the treatment of tetanus. 6. Serum treatment is useful as a preventive, and also in the developed disease when it is possible to apply it early or when the production of toxins is still going on. But the results of this method, if they cannot be ignored, are neither convincing nor brilliant. Even if it were the most efficacious method, symptomatic treatment must not be neglected. 7. A patient suffering from tetanus must be treated carefully, regard being had to the wound, to the intensity of the intoxication and its duration, and the special conditions present in the case. Carbolic acid in a large measure fulfils the indications, and is therefore suitable for the majority of cases."

Dr. D. F. Woods (*New York Medical Journal*) has also reported a case of tetanus treated with carbolic acid, and states that this case was the only one he had ever seen recover. He concludes that this agent to be effective must be given in large quantities and heroic doses, and in such is much more reliable than any serum he has ever tried. He also reports a case of tetanus in a horse being cured by the use of this agent at his suggestion.—*Ephemeris.*

Apomorphine Counteracted by Boric Acid. Dr. C. J. Douglas reports (*New York Medical Journal*) that apomorphine when dissolved in a concentrated solution of boric acid is completely neutralized both as to its emetic and hypnotic properties. This appears to be the first mention of this important fact, and recalls the somewhat similar influence of boric acid on cocaine.

Borax as a General Poison Antidote. Crouzel (*Progressive Medicine*) suggests that borax dissolved in twenty parts of milk would constitute one of the best general antidotes in cases of poisoning. Most mineral bases, he points out, are precipitated by boric acid as insoluble caseinates with mineral bases ; toxic acids cause coagulation of the casein ; and, lastly, the fat of the milk in a measure protects the mucous lining against irritants.

The treatment of injured flexion surfaces should always be supplemented by the application of appropriate splints to prevent movement during the healing process.—*Id.*

